# Potential Role for Diet in Mediating the Association of Olfactory Dysfunction and Cognitive Decline: A Nationally Representative Study

**DOI:** 10.3390/nu15183890

**Published:** 2023-09-07

**Authors:** Varun Vohra, Sahar Assi, Vidyulata Kamath, Zachary M. Soler, Nicholas R. Rowan

**Affiliations:** 1 Department of Otolaryngology-Head and Neck Surgery, School of Medicine, Johns Hopkins University, Baltimore, MD 21287, USA; vvohra1@jhmi.edu; 2 Cochlear Center for Hearing and Public Health, Johns Hopkins University, Baltimore, MD 21205, USA; sassi1@jhu.edu; 3 Department of Epidemiology, Johns Hopkins Bloomberg School of Public Health, Baltimore, MD 21205, USA; 4 Department of Psychiatry and Behavioral Sciences, School of Medicine, Johns Hopkins University, Baltimore, MD 21287, USA; vkamath@jhmi.edu; 5 Department of Otolaryngology-Head & Neck Surgery, Medical University of South Carolina, Charleston, SC 29425, USA; solerz@musc.edu; 6 Department of Neurosurgery, School of Medicine, Johns Hopkins University, Baltimore, MD 21287, USA

**Keywords:** olfaction, diet, cognition

## Abstract

In the context of a growing body of evidence associating olfactory dysfunction (OD) with cognitive decline, this cross-sectional study used data from the 2013–2014 National Health and Nutrition Examination Survey (NHANES) sample in order to explore the role of dietary intake in this association. Leveraging a nationally representative sample of U.S. adults aged 60 years and older, this study analyzed dietary patterns using exploratory factor analysis. OD was categorized based on the NHANES Pocket Smell Test, and cognitive function was measured with a battery of tests. Survey-weighted multivariable regressions and causal mediation analysis were used to examine the relationship between dietary patterns, OD, and cognitive function. Results indicated that a poor adherence to a diet rich in monounsaturated fats (MUFAs) and polyunsaturated fats (PUFAs) was independently associated with both cognitive and olfactory dysfunctions, after adjusting for sociodemographic and health factors. Moreover, the relationship between OD and cognitive decline was found to be partly mediated by adherence to such a diet. This study proposes a potential link between diet, olfactory function, and cognitive decline, highlighting the role of nutritional interventions in mitigating cognitive decline, particularly in individuals with olfactory impairment.

## 1. Introduction

Olfactory dysfunction (OD) is a highly prevalent condition among adults, affecting 13.5% of adults in the U.S. and up to 25% worldwide [1,2]. OD increases in prevalence with aging such that up to 62.5% of adults over the age of 80 have OD [3,4]. Mounting evidence has linked OD to cognitive decline and neurodegenerative diseases, although the underlying mechanisms are not fully understood [5,6,7,8].

One potential pathway linking olfaction to cognition is nutritional intake. OD may alter the perception of flavor and subsequently affect food selection and quality, although evidence is inconsistent [9,10,11,12]. Previous single-nutrients studies investigating variations in diet found lower intake of fat, protein, sodium, and potassium among adults with OD [13,14]. Importantly, certain nutrients and diets, such as omega-3 fatty acids and a Mediterranean diet, may play a preventative role in cognitive decline and Alzheimer’s dementia (AD) [15,16,17]. Additionally, one study found that supplementing APOE4 transgenic mice with omega-3 fatty acids improved both olfactory and cognitive function, highlighting a potential intersection between diet, olfaction, and cognition [18].

Although prior studies have established that OD is associated with both dietary changes and cognitive decline, the role of diet in the association of OD and cognition has not been previously explored. In addition, few studies linking diet to olfaction and cognition have used data-driven approaches such as principal components analysis (PCA) and exploratory factor analysis (EFA) to characterize dietary patterns, which provide a more accurate representation of diet compared to single-nutrients analyses [19]. Therefore, the objectives of this study were (1) to characterize PCA-derived dietary patterns in a nationally representative sample of adults and examine their association with OD, (2) to analyze the independent associations of OD and dietary patterns with cognitive function, and (3) to explore the role of dietary patterns in the association of OD with cognitive function through a mediation analysis. We hypothesize that dietary changes contribute to the association of OD and cognitive decline.

## 2. Materials and Methods

### 2.1. Population

The study population for this analysis were subjects from the 2013–2014 National Health and Nutrition Examination Survey (NHANES) cycle aged 60 years and older who completed psychophysical olfactory testing, dietary interviews, and had complete data for all examined covariates.

### 2.2. Olfactory Assessment

The 2013–2014 NHANES assessed psychophysical olfactory functioning with the 8-item Pocket Smell Test (PST, Sensonics, Inc., Haddon Heights, Camden County, NJ, USA) as previously described [1,3]. Briefly, the test assessed odor identification ability with eight suprathreshold separate odors presented in a forced-choice multiple choice format. Participants were asked to identify each odor from a list of four descriptors. Tests were scored on a scale from 0 to 8 based on the sum of correct responses. Participants who identify 3 or more out of 8 odorants incorrectly were considered to have OD.

### 2.3. Dietary Recall 

The 2013–2014 NHANES contained a dietary intake interview that assessed dietary intake on three nonconsecutive 24-h periods. A registered dietician provided guidance on maintaining a detailed food record. The “Total Nutrient Intakes” files from NHANES 2013–2014 were used. 24-h recalls included data on estimated consumption of macronutrients (total protein, carbohydrates, and fats) and micronutrients (vitamins and minerals). Dietary components examined in this study are listed in Table S1. Nutrient data and dietary data-specific sample weights for each dietary interview day were averaged for all survey-adjusted statistical analyses.

### 2.4. Cognitive Assessments

Cognitive evaluations included the Consortium to Establish a Registry for Alzheimer’s Disease (CERAD) battery, the Digit Symbol Substitution Test (DSST), and the Animal Fluency Test (AF) [20,21,22]. The CERAD word learning (CERAD-WL) and CERAD Delayed Word Recall (CERAD-DR) were used to assess the ability to encode and recall verbal information, respectively. Testing consisted of vocalization and subsequent recall of a set of ten unrelated words across three learning trials, with CERAD-WL scores ranging from 0 to 30. After a delay, corresponding to completion of other cognitive assessments, CERAD-DR was conducted, with scores ranging from 0 to 10. The DSST was used to assess focal attention, associative learning, and information processing speed. Participants were asked to correctly match a sequence of symbols to their corresponding numbers and were scored based on the number of accurately completed pairings within a two-minute interval. Lastly, the AF test was used to measure category-guided word generativity. Participants were required to articulate as many animal names as possible within a single minute, allocating a single point for each unique animal mentioned.

### 2.5. Statistical Analysis

Statistical analyses were conducted using R statistical software (version 4.3.1, https://www.r-project.org/, accessed on 25 June 2023). All statistical analysis followed CDC guidelines for complex, multistage survey analysis in NHANES, including the appropriate weights for all analyses.

In descriptive analyses of the analytic sample stratified by olfactory function (OD vs. no OD), we compared categorical variables using survey-weighted chi-square tests with the Scott–Rao correction (expressed as counts and proportions) and continuous variables using survey-weighted Kruskal–Wallis tests (expressed as means and standard deviations).

Exploratory factor analysis was used to derive dietary patterns (DPs), employing the principal component extraction method. The suitability of the nutrient data for factor analysis was verified with the Kaiser–Meyer–Olkin (KMO) test, which showed a value of 0.82, indicating satisfactory sample adequacy. Bartlett’s test of sphericity was also performed (*p* < 0.001), confirming a correlation between nutrients. Parallel analysis and visual examination of the scree plot were used for selection of the number of factors. An orthogonal varimax rotation was performed on the initial factor solution to simplify the structure and enhance its interpretability. Adherence scores, also known as factor scores, were determined as the linear combination of factor loadings and standardized nutrient intakes for each DP. Higher scores are indicative of stronger adherence to a particular DP. DPs were named based on the two highest factor loadings, and these names were used for reference throughout the text.

For bivariate and multivariate analyses, pattern adherence scores for each DP were categorized into quartiles with the 4th quartile (Q4) indicating the highest adherence and the 1st quartile (Q1) indicating the poorest adherence. For these analyses, the reference group was Q4. Bivariate linear regression models were used to plot PST scores across quartiles of each DP. Adjusted logistic regression models were used to assess the association between DPs and OD. Adjusted linear regression models were used to assess the association between OD and cognition and DPs and cognition. Lastly, causal mediation analyses were conducted to investigate the mediation of dietary patterns in the association of OD and cognition. DPs were analyzed continuously in these analyses. The total effect of OD was divided into a direct and indirect effect, the latter attributed to the mediation variable. We reported the average direct effect (ADE) attributed to OD, the average causal mediation effect (ACME) attributed to diet, and the total effect on cognitive test scores. In all analyses, nutrient intakes were adjusted for energy using the residual method. Covariates in adjusted models included age, gender, race, income-to-poverty ratio, education, body mass index (BMI), total energy intake, smoking status, history of head/face injury, and history of persistent cold/flu symptoms.

## 3. Results

### 3.1. Participant Characteristics

A total of 1310 participants from NHANES were included in our analytic sample. Among them, 285 (21.8%) had OD on testing while 1025 (78.2%) did not. Relative to those without OD, participants with OD were older on average (72.06 ± 7.19 vs. 68.80 ± 6.37 years, *p* < 0.001) and more likely to be male (59.9% vs. 46.7%, *p* < 0.001). Additionally, participants with OD had a relatively lower income-to-poverty ratio (2.49 ± 1.47 vs. 2.75 ± 1.62, *p* = 0.02) and were less likely to have a high school diploma (41.1% vs. 57.3%, *p* < 0.001). They also had lower average daily caloric intake (1751 ± 673 vs. 1864 ± 679, *p* = 0.013) (Table 1).

### 3.2. Dietary Patterns and Olfaction

**Figure 1:**
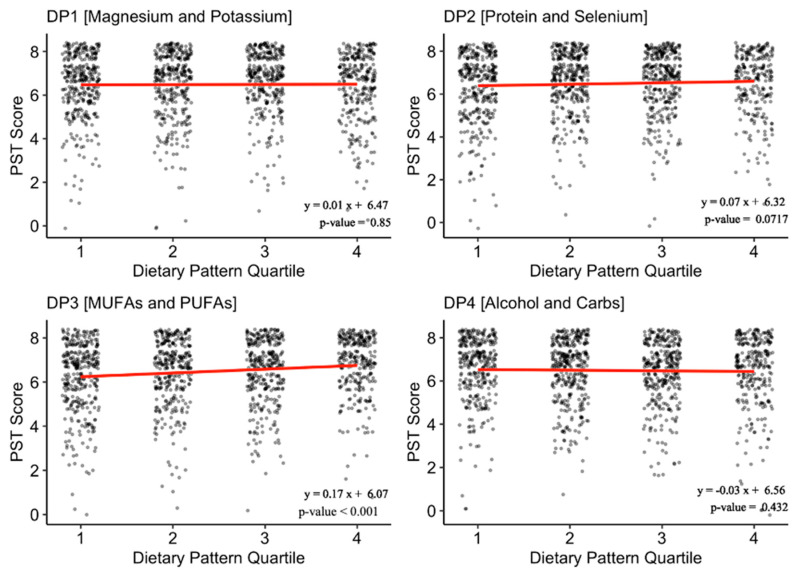
Association of Pocket Smell Test Scores with Adherence to Dietary Patterns. Footnotes: Unadjusted linear regression model of PST score across quartiles of adherence to the derived dietary patterns. Higher PST score indicates better olfaction. Higher DP quartile indicates better adherence to the DP. Abbreviations: MUFAs: monounsaturated fatty acids; PST: pocket smell test; PUFAs: polyunsaturated fatty acids.

Factor analysis yielded 4 dietary patterns (DPs) from 30 nutrition variables: DP1 (Magnesium and Phosphate), DP2 (Protein and Selenium), DP3 (monounsaturated fatty acids (MUFAs) and polyunsaturated fatty acids (PUFAs)), and DP4 (Alcohol and Carbs) (Table S1). Together, these DPs accounted for 46% of the variance in the sample dietary data. On bivariate analysis examining the association between OD and adherence scores to each DP, only adherence to DP3 (MUFAs and PUFAs) significantly differed between participants with OD and without OD (Table 1). Additional linear regression analysis confirmed that DP3 was significantly and positively correlated with PST score (β = 0.17 (0.11, 0.23), *p* < 0.001) (Figure 1). Fully adjusted multivariable logistic regression examining the odds of having OD across quartile groups of adherences to DP3 indicated that relative to participants with high adherence (Q4), those with poorer adherence had greater odds of having OD (Q1, OR = 2.11 (1.39, 3.18), *p* = 0.003) (Table 2).

### 3.3. Olfaction, Dietary Patterns, and Cognition: Multivariable Analysis

On bivariate analysis, OD was associated with lower scores across the four cognitive tests (CERAD-WL, 17.88 ± 4.87 vs. 20.39 ± 4.28; CERAD-DR, 5.38 ± 2.45 vs. 6.62 ± 2.12; DSST, 39.52 ± 15.91 vs. 49.95 ± 15.98; and AF, 14.94 ± 4.83 vs. 17.58 ± 5.44, *p* < 0.001 for all) (Table 1). In multivariable linear regression models including both OD and DP3, we found that participants with OD scored significantly lower on the CERAD-WL (β = −1.77 (−2.79, −0.79), *p* = 0.006), CERAD−DR (β = −0.93 (−1.52, −0.35), *p* = 0.005), DSST (β = −5.56 (−10.2, −0.88), *p* = 0.028), and AF (β = −2.06 (−3.7, −0.41), *p* = 0.013) cognitive testing compared to participants without OD (Table 3). Lower adherence to DP3 was similarly associated with significantly worse performance across all cognitive tests. Specifically, relative to the highest quartile of adherence, being in the lowest quartile of adherence was associated with a decrease in score on the CERAD-WL (β = −1.66(−2.7, −0.62), *p* = 0.009), CERAD-DR (β = −0.57(−1.12, −0.013), *p* = 0.045), DSST (β = −4.31(−7.38, −1.23), *p* = 0.013), and AF (β = −1.82 (−3.4, −0.26), *p* = 0.03) (Table 3).

### 3.4. Olfaction, Dietary Patterns, and Cognition: Causal Mediation Analysis

**Figure 2:**
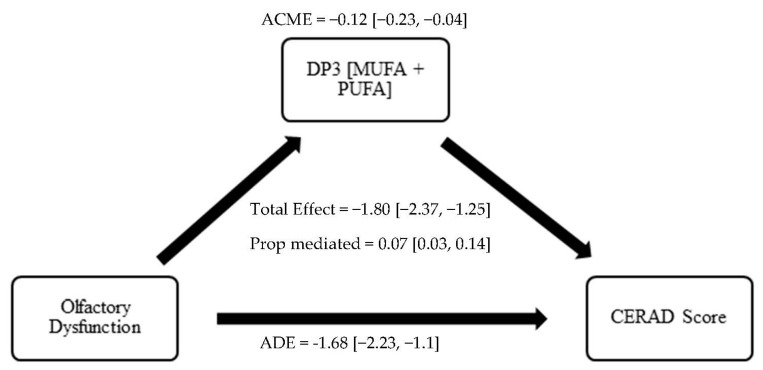
Mediation of the Association between OD and cognitive functioning by Dietary Pattern 3 (Monosaturated Fatty Acids and Polysaturated Fatty Acids). Abbreviations: ACME, average causal mediation effect; ADE, average direct effect; Proportion mediated = ACME/Total effect; CI, confidence interval; DP3, Dietary Patter 3; PUFA, polyunsaturated fatty acid; MUFA, monounsaturated fatty acid.

Results of the causal mediation analysis investigating the role of adherence to DP3 in the association between OD and cognition are shown in Table 4 (Models 1–4). We found that both direct and indirect effects played a significant role in the association of OD with lower cognitive scores. On CERAD-WL testing, the total effect of OD was a −1.80 ((−2.37, −1.25), *p* < 0.001), the ADE was −1.68 ((−2.23, −1.1), *p* < 0.001), the ACME was −0.12 ((−0.23, −0.04), *p* < 0.001), and the proportion of the effect mediated was 0.07 ((0.03, 0.14), *p* < 0.001) (detailed in Figure 2). On CERAD-DR testing, the proportion of the effect mediated was 0.05 ((0.02, 0.12), *p* = 0.003). On DSST testing, the proportion of the effect mediated was 0.08 ((0.04, 0.14), *p* < 0.001). Lastly, on AF testing, the proportion of the effect mediated was 0.06 ((0.02, 0.12), *p* = 0.008).

## 4. Discussion

In this cross-sectional analysis of a nationally representative sample of U.S. adults aged 60 years and older, we found that poor adherence to a diet high in MUFAs and PUFAs was independently associated with olfactory and cognitive dysfunction after adjusting for sociodemographic and health covariates. Importantly, using a mediation analysis, we found that poor adherence to a diet high in MUFAs and PUFAs played a significant role in the association of OD and cognitive decline. While the relationship between OD and cognitive decline is likely multifactorial, our findings highlight that dietary changes associated with OD may play a role in the well-established association between OD and cognitive dysfunction.

Our findings are in line with a large body of evidence demonstrating a strong association between olfactory dysfunction and cognitive decline. This association, indicating the role of OD as biomarker of neurodegenerative disease, has been previously established in several epidemiological studies [5,6,7,8,23,24]. Notably, this association between OD and cognitive function has been previously shown within the 2013–2014 NHANES sample, and the current study findings confirm this association in our subsample including dietary data [5].

Additionally, there has been growing interest in recent studies on the role of diet in the prevention or delay of cognitive decline and dementia [25]. Several prospective cohort studies have consistently found that intake of unsaturated fats is associated with reduced risk of cognitive decline and dementia over time [26,27]. Suggested mechanisms include atherosclerotic disease, with studies having shown that a high ratio of saturated to unsaturated fats is associated with a poor cholesterol profile, and consequently, an increased risk of Alzheimer’s dementia [25,28]. Other hypothesized mechanisms relate to the role of fatty acids in maintaining the structural integrity of neuronal membranes, modifying the function of neurotransmitter receptors, and facilitating cellular membrane proteins [27,29,30,31].

Notably, there is limited prior investigation of the role of diet in the association between olfactory dysfunction and cognitive decline. As a chemosensory function, olfactory dysfunction expectedly has an impact on dietary patterns. Numerous studies have found that OD is associated with poorer diet quality and intake of certain nutrients, including monounsaturated and polyunsaturated fats, leading to poorer nutritional status [11,13,14,32,33]. Interestingly, a randomized controlled trial by Yan et al. demonstrated a protective role of Omega-3 fatty acid supplementation for post-operative olfactory function after endoscopic sinus surgery, underscoring the impact of diet on olfactory function [34].

Our novel approach on disentangling the relationship of OD and cognitive decline suggests an intriguing role of fatty acid intake in this association. One likely underlying mechanism involves chronic inflammation. Indeed, prior studies of OD, cognitive decline, and dietary patterns have independently identified associations with chronic inflammation [35,36,37]. Specifically, established diets high in MUFA/PUFA, including the Mediterranean diet, have been shown to have an anti-inflammatory effect [37]. While inflammation is likely to play an important role, alternative mechanisms are also plausible. For example, dysfunction in the olfactory system may lead to changes in endocrine regulation, metabolism, and energy homeostasis [38]. Interestingly, one study found that a large proportion of obese patients recovered their olfactory function after undergoing bariatric surgery, further highlighting the link between metabolic changes and OD [39].

Several limitations should be considered in this study. Primarily, the cross-sectional nature of the study design limits our ability to determine the direction of causality and mediation between OD, dietary changes, and cognitive decline. Additionally, characterized dietary patterns were based on self-reported survey-collected dietary data and may be subject to bias. Furthermore, although we adjusted for multiple confounders in our analysis, there may be residual unmeasured confounding in the studied associations. Lastly, the results of this analysis indicate that the effect size of dietary changes associated with OD are limited to 5–10% of the total effect size. Therefore, our findings require additional longitudinal studies and replication to investigate the clinical significance of nutritional interventions. Despite these limitations, we believe this study offers exciting new evidence that, in individuals with OD, a diet high in monounsaturated and polyunsaturated fats may have a protective effect on cognition.

## 5. Conclusions

In a nationally representative sample of adults aged 60 and older in the U.S., we found that OD was associated with poor adherence to a diet high in MUFAs and PUFAs, and that both this dietary pattern and OD were independently associated with poorer cognitive function. Additionally, we found that the association of OD with poorer cognitive function was partly mediated by poor adherence to a diet high in MUFAs and PUFAs. Our results highlight the potential mediating role of diet in the association between OD and cognitive decline.

## Figures and Tables

**Table 1:** Demographics, Cognitive Function, and Dietary Patterns by OD Status Among Adults from the National Health and Nutrition Examination Survey (NHANES), 2013–2014.

	Overall	No OD	OD	*p*-Value
Number of observations: n (%)	1310 (100)	1025 (78.2)	285 (21.8)	
Age: mean (sd)	69.51 (6.69)	68.80 (6.37)	72.06 (7.19)	<0.001 ***
Gender: n (%)				<0.001 ***
Male	626 (47.8)	458 (46.7)	168 (59.9)	
Female	684 (52.2)	567 (55.3)	117 (41.1)	
Race: n (%)				0.129
Non-Hispanic White	698 (53.3)	559 (54.5)	139 (48.8)	
Mexican American	140 (10.7)	114 (11.1)	26 (9.1)	
Other Hispanic	106 (8.1)	75 (7.3)	31 (10.9)	
Non-Hispanic black	265 (20.2)	202 (19.7)	63 (22.1)	
Non-Hispanic Asian	87 (6.6)	63 (6.1)	24 (8.4)	
Other	14 (1.1)	12 (1.2)	2 (0.7)	
Education: n (%)				<0.001 ***
≤High School Diploma	594 (45.3)	438 (42.7)	168 (58.9)	
>High School Diploma	716 (54.7)	587 (57.3)	117 (41.1)	
Income-to-Poverty ratio: mean (sd)	2.69 (1.59)	2.75 (1.62)	2.49 (1.47)	0.020 *
BMI: mean (sd)	29.17 (6.26)	29.33 (6.29)	28.59 (6.16)	0.079
Current Smoker: n (%)				0.990
Yes	145 (11.1)	114 (11.1)	31 (10.9)	
No	2433 (0.83)	911 (88.9)	254 (89.1)	
Self-reported OD: n (%)				<0.001 ***
Yes	251 (19.2)	170 (16.6)	81 (28.4)	
No	1059 (81.8)	855 (83.4)	204 (79.6)	
History of Head/Face Injury				0.99
Yes	179 (13.7)	140 (13.7)	39 (13.7)	
No	1131 (86.3)	885 (86.3)	246 (86.3)	
History of Persistent Cold/Flu in last 12 months				0.95
Yes	86 (6.6)	68 (6.6)	18 (6.3)	
No	1224 (93.4)	957 (93.4)	267 (93.7)	
Cognitive Function				
CERAD-WL: mean (sd)	19.85 (4.53)	20.39 (4.28)	17.88 (4.87)	<0.001 ***
CERAD-DR: mean (sd)	6.35 (2.25)	6.62 (2.12)	5.38 (2.45)	<0.001 ***
DSST: mean (sd)	47.68 (16.53)	49.95 (15.98)	39.52 (15.91)	<0.001 ***
Animal Fluency: mean (sd)	17.01 (5.42)	17.58 (5.44)	14.94 (4.83)	<0.001 ***
Dietary Data				
Energy (kcal): mean (sd)	1839.18 (679.25)	1864 (679)	1751 (673)	0.013 *
DP1 (Magnesium and Potassium): n (%)				0.111
Q1	345 (26.3)	269 (26.2)	76 (26.7)	
Q2	338 (25.8)	257 (25.1)	81 (28.4)	
Q3	332 (25.3)	275 (26.8)	57 (20.0)	
Q4	295 (22.5)	224 (21.9)	71 (24.9)	
DP2 (Protein and Selenium): n (%)				0.116
Q1	351 (26.8)	259 (25.3)	92 (32.3)	
Q2	355 (27.1)	287 (28.0)	68 (23.9)	
Q3	347 (26.5)	275 (26.8)	72 (25.3)	
Q4	257 (19.6)	204 (19.9)	53 (18.6)	
DP3 (MUFA and PUFA): n (%)				0.002 **
Q1	350 (26.7)	253 (24.7)	97 (34.0)	
Q2	332 (25.3)	258 (25.2)	74 (26.0)	
Q3	338 (25.8)	268 (26.1)	70 (24.6)	
Q4	290 (22.1)	246 (24.0)	44 (15.4)	
DP4 (Alcohol and Carbs): n (%)				0.786
Q1	329 (25.1)	262 (25.6)	67 (23.5)	
Q2	357 (27.3)	273 (26.6)	84 (29.5)	
Q3	340 (26.0)	267 (26.0)	73 (25.6)	
Q4	284 (21.7)	223 (21.8)	61 (21.4)	

* *p*-value < 0.05 ** *p*-value < 0.005 *** *p*-value < 0.0005. Abbreviations: CERAD: Consortium to Establish a Registry for Alzheimer’s Disease; CERAD-WL: CERAD word learning; CERAD-DR: CERAD delayed word recall; DP: dietary pattern; DSST: Digit Symbol Substitution Test; MUFA: monounsaturated fatty acids; OD: olfactory dysfunction; PUFA: polyunsaturated fatty acids.

**Table 2:** Association of Adherence to Dietary Pattern 3 (Monosaturated Fatty Acids and Polysaturated Fatty Acids) with Odds of Olfactory Dysfunction.

Odds of Olfactory Dysfunction			
	OR	95% CI	*p*-Value
Quartiles of adherence to DP3 *			
Q4	ref	-	-
Q3	1.73	(0.75, 3.98)	0.16
Q2	1.51	(0.89, 2.56)	0.1
Q1	2.11	(1.39, 3.18)	0.003 **

* Q4 (reference group) indicates high adherence to DP3, whereas Q1 indicates poor adherence. Estimates from a multivariate logistic regression model adjusted for age, gender, race, education, income-to-poverty ratio, body mass index, total energy intake, smoking status, history of head/face injury, and history of persistent flu/cold. ** *p*-value < 0.005. Abbreviations: DP3: dietary pattern 3; CI: confidence interval; OR: odds ratio.

**Table 3:** Association of Olfactory Dysfunction and Adherence to Dietary Pattern 3 (Monosaturated Fatty Acids and Polysaturated Fatty Acids) with Cognitive Test Scores.

	Difference in Cognitive Test Scores		
	β-Estimate	95% CI	*p*-Value
CERAD-WL			
OD	−1.77	(−2.79, −0.79)	0.006 **
DP3			
Q4	ref	-	-
Q3	−0.08	(−0.88, 0.72)	0.8
Q2	−0.88	(−1.89, 0.13)	0.076
Q1	−1.66	(−2.7, −0.62)	0.009**
CERAD-DR			
OD	−0.93	(−1.52, −0.35)	0.005 **
DP3			
Q4	ref	-	-
Q3	−0.17	(−0.69, 0.35)	0.46
Q2	−0.42	(−0.9, 0.05)	0.07
Q1	−0.57	(−1.12, −0.013)	0.045 *
DSST			
OD	−5.56	(−10.2, −0.88)	0.026 **
DP3			
Q4	ref	-	-
Q3	0.96	(−3.32, 5.25)	0.61
Q2	−2.46	(−5.7, 0.82)	0.12
Q1	−4.31	(−7.38, −1.23)	0.013 *
AF			
OD	−2.06	(−3.7, −0.41)	0.023 *
DP3			
Q4	ref	-	-
Q3	0.19	(−0.71, 1.55)	0.61
Q2	−0.1	(−1.23, 1.44)	0.85
Q1	−1.82	(−3.4, −0.26)	0.03 *

Estimates from a multivariate linear regression model adjusted for age, gender, race, income-to-poverty ratio, body mass index, total energy intake, and smoking status. * *p*-value < 0.05 ** *p*-value < 0.005. Abbreviations: CERAD: Consortium to Establish a Registry for Alzheimer’s Disease; CERAD-WL: CERAD word learning; CERAD-DR: CERAD delayed word recall; CI: confidence interval; DSST: Digit Symbol Substitution Test.

**Table 4:** Mediation and Multivariable Analyses of association between and OD and cognitive functioning by Adherence to Dietary Pattern 3 (Monosaturated Fatty Acids and Polysaturated Fatty Acids).

	Mediation Analysis	
	Beta (95% CI)	*p*-Value
CERAD-WL		
Model 1		
ACME	−0.12 (−0.23, −0.04)	<0.001 ***
ADE	−1.68 (−2.23, −1.1)	<0.001 ***
Total Effect	−1.80 (−2.37, −1.25)	<0.001 ***
Prop. Mediated	0.07 (0.03, 0.14)	<0.001 ***
CERAD-DR		
Model 2		
ACME	−0.05 (−0.09, −0.01)	0.004 **
ADE	−0.99 (−1.27, −0.72)	<0.001 ***
Total Effect	−1.04 (−1.32, −0.78)	<0.001 ***
Prop. Mediated	0.05 (0.02, 0.12)	0.003 **
DSST		
Model 3		
ACME	−0.35 (−0.55, −0.16)	<0.001 ***
ADE	−3.99 (−6.01, −1.9)	<0.001 ***
Total Effect	−4.34 (−6.44, −2.23)	<0.001 ***
Prop. Mediated	0.08 (0.04, 0.14)	<0.001 ***
AF		
Model 4		
ACME	−0.14 (−0.26, −0.04)	0.008 **
ADE	−2.05 (−2.75, −1.36)	<0.001 ***
Total Effect	−2.19 (−2.9, −1.49)	<0.001 ***
Prop. Mediated	0.06 (0.02, 0.12)	0.008 **

Models were adjusted for age, gender, race, income-to-poverty ratio, education, body mass index, total energy intake, and smoking status. In these analyses, DP3 was modeled continuously. ** *p*-value < 0.005 *** *p*-value < 0.0005. Abbreviations: CERAD: Consortium to Establish a Registry for Alzheimer’s Disease; CERAD-WL: CERAD word learning; CERAD-DR: CERAD delayed word recall; CI: confidence interval; DSST: Digit Symbol Substitution Test.

## Data Availability

The publicly available National Health and Nutrition Examination Survey (NHANES) 2013–2014 database was used in this study, which may be found here: https://wwwn.cdc.gov/nchs/nhanes/search/DataPage.aspx?Component%20=%20Question-naire&CycleBeginYear%20=%202013 (accessed on 25 June 2023).

## Supplementary Materials

The following supporting information can be downloaded at: https://www.mdpi.com/article/10.3390/nu15183890/s1, Table S1. Factor Loadings of PCA-derived Dietary Patterns.
