# Supplementary material for: Potential Role for Diet in Mediating the Association of Olfactory Dysfunction and Cognitive Decline: A Nationally Representative Study
Source: Nutrients. 2023 Sep 7;15(18):3890. doi: 10.3390/nu15183890 (PMC10538071; doi:10.3390/nu15183890)
Supplement: Supplementary file 1 [file nutrients-15-03890-s001.zip › nutrients-2551287-supplementary.pdf]

Supplementary Table

Table S1. Factor Loadings of PCA-derived Dietary Patterns.

**Dietary Components**	**Magnesium and Potassium DP (17.5%)**	**Protein and Selenium DP (11.5%)**	**MUFA and PUFA DP (11.2 %)**	Alcohol and Carbs DP (5.8%)
Protein	0.23	0.97	0	-0.05
Carbs	0.17	-0.47	-0.56	0.66
Total sugar	-0.01	-0.38	-0.34	0.51
Fiber	0.74	-0.06	-0.09	0.12
Total Fat	-0.15	0.04	0.99	-0.01
Saturated Fat	-0.3	0.02	0.68	0.06
MUFA	-0.06	0.05	0.87	-0.07
PUFA	0.07	-0.06	0.76	-0.04
Cholesterol	-0.16	0.51	0.21	-0.09
Vitamin A	0.57	0	-0.02	0.03
Beta-Carotene	0.49	0.03	-0.06	-0.03
Vitamin B1	0.39	0.11	-0.18	0.16
Vitamin B2	0.45	0.16	-0.05	0
Niacin	0.33	0.52	-0.14	-0.03
Vitamin B6	0.51	0.31	-0.17	-0.02
Folate	0.24	-0.11	-0.17	0.19
Vitamin C	0.49	-0.01	-0.14	0.02
Vitamin D	0.31	0.29	-0.04	0.02
Vitamin K	0.43	0.08	0.1	-0.12
Calcium	0.42	0.13	0.02	0.08
Phosphorous	0.5	0.61	0.03	-0.01
Magnesium	0.84	0.15	-0.03	-0.13
Iron	0.48	0	-0.14	0.15
Zinc	0.41	0.4	0.03	0.01
Copper	0.66	0.07	0.04	0
Sodium	0.06	0.48	0.02	0.05
Potassium	0.76	0.26	-0.12	-0.03
Selenium	0.1	0.7	0	-0.02
Caffeine	0.04	-0.04	0.03	-0.06
Alcohol	-0.15	-0.12	-0.29	-0.94

MUFA = monounsaturated fatty acid, PUFA = polyunsaturated fatty acids
